# Early outcome of Frey’s procedure for chronic pancreatitis: Nepalese tertiary center experience

**DOI:** 10.1186/s12893-019-0592-7

**Published:** 2019-09-18

**Authors:** Dhruba Narayan Sah, Ramesh Singh Bhandari, Yogendra Prasad Singh, Pradeep Vaidya, Prasan B. S. Kansakar, Bikal Ghimire, Bishnu Kandel, Jayant Kumar Sah, Paleswan Joshi Lakhey

**Affiliations:** Department of GI & General Surgery, Institute of Medicine, Tribhuvan University Teaching Hospital, Maharajgunj, P. O - 1524, Kathmandu, 00977 Nepal

**Keywords:** Chronic pancreatitis, Surgery, Frey’s procedure, Izbicki, Nepal

## Abstract

**Background:**

Chronic pancreatitis is a progressive and persistent inflammatory disease resulting in pancreatic insufficiency leading to diabetes and steatorrhea. Abdominal pain is the most debilitating feature and is often refractory to treatment. Medical management with adequate analgesia and replacement of pancreatic enzyme supplements is the first line in management of chronic pancreatitis. Surgery is reserved for those who fail medical management. The choice of surgical procedure and timing of surgery is a topic of debate.

The objective of this study was to analyze surgical safety along with short- and long- term outcomes of Frey’s procedure for patients suffering from chronic pancreatitis.

**Methods:**

This was a retrospective review of cases of chronic pancreatitis who underwent Frey’s procedure from 2016 January to 2019 February at Tribhuvan University Teaching Hospital. Demographics, intraoperative findings, perioperative outcomes, and short- and long-time outcomes were analyzed.

**Results:**

Total of 26 patients (age ranged 17–52, male − 14) underwent Frey’s procedure in the study period. Alcohol was etiology in six patients while the majority (76.9%) were nonalcoholic. Half of the patients had tropical pancreatitis. Intractable pain was present in all cases along with pseudocyst in three and pseudoaneurysm in one case. The mean preoperative Izbicki scores were 53.4 ± 17.6. Six patients had diabetes and two patients had steatorrhea. Major complications were seen in 11.5% of cases while mortality was in one patient. The median duration of the hospital stay was seven days. Over a median follow up of 17 months (range, 3–38), there were significantly lower pain scores postoperatively and 92% were pain-free. Only one new case of diabetes developed postoperatively.

**Conclusion:**

Our early experiences suggests that Frey’s procedure can be a safe option for patients with chronic pancreatitis, with acceptable perioperative morbidity with adequate pain relief without worsening of pancreatic endocrine and exocrine function.

## Background

Chronic pancreatitis (CP) is defined as an inflammatory disease of the pancreas characterized by persistent and often progressive fibrosis and irreversible morphological changes, leading to epigastric pain and/or exocrine and endocrine insufficiency [1, 2]. Classical triad of CP includes abdominal pain, exocrine pancreatic insufficiency (steatorrhea, weight loss, deficiency of fat-soluble vitamins,) and diabetes. Abdominal pain is the most frequent and debilitating symptom with a variable pattern [3]. Pain is often refractory to treatment, with a frequent hospital visit, psychosocial problems, opioids dependence [3]. Tropical pancreatitis is a separate entity whose etiology is not well defined and usually, the patients belong to the tropical zone of Nepal and India.

The management of CP requires multidisciplinary approach involving pain management specialists, gastroenterologist, radiologist, surgeons, dietitian and psychiatrists. Gastroenterologists usually opt for endoscopic treatment before considering surgical treatment as it is less invasive and without major complications. Endoscopic treatment is warranted in patients with CP who have intraductal stones in the region of the pancreatic head, main pancreatic duct (MPD) stricture, and symptomatic pseudocyst [3].

As endoscopic treatment requires frequent hospital admissions, inadequate pain relief, increasing the risk opioids dependence and most importantly rural located populations, surgical option was preferred more often as compared to repeated endoscopic therapy. Nepal has a great geographical diversity and health system in outside of capital (Kathmandu) is yet to develop properly. It is crucial to understand that patients from rural areas won’t come for repeat endoscopy and therefore surgery was chosen at first hand.

Surgical treatment in CP includes either resection or drainage or hybrid procedures. The degree and extent of MPD dilatation along with gland morphology will guide in choosing the optimal operative procedure. A metanalysis showed Frey’s procedure had shorter operative duration along with less overall perioperative complications as compared to pancreatoduodenectomy (PD) and the Beger procedure [3, 4]. Also patients who underwent Frey’s procedure had a more favorable quality of life and pancreatic function outcomes [4]. Many studies have shown excellent long term outcome with surgical treatment [5]. Though optimal timing of surgery is still a topic of debate, many surgeons advocate early operation because of superiority in achieving pain relief and improvement of quality of life [6, 7]. Frey’s procedure can be a standard treatment for CP with severe inflammation in the pancreatic head with a dilated duct.

The main objective of this study was to evaluate the safety and outcome of the Frey’s procedure for patients with CP at Tribhuvan University Teaching Hospital (TUTH), a Nepalese tertiary referral center.

## Methods

This was a retrospective review of prospectively collected data of surgically treated cases of CP who underwent Frey’s procedure from January 2016 to February 2019 at the department of surgical gastroenterology, Tribhuvan University Teaching Hospital (TUTH), Kathmandu, Nepal. All patients who underwent Frey’s procedure and had minimum of 3 months of follow-up available were included in this study. Exclusion criteria were procedure other than Frey’s procedure, lost to follow-up and inadequate data. Total of 38 cases of CP was operated in the above-mentioned duration out of which 26 underwent Frey procedure. Besides Frey, other surgical procedures were Partington- Rochelle procedure (*n* = 4), Beger with Bern modification (*n* = 1) and PD (*n* = 7).

Surgery is usually indicated in patients who failed medical & endoscopic therapy, developed complications or if suspicious of malignancy. At our institute choice of surgical procedure depends on ductal morphology, pancreatic head enlargement, suspicious of malignancy and associated locoregional complications. Choice of Frey procedure was based on MPD > 7 mm along with pancreatic head diameter > 4 cm as suggested by others in the literature [8]. Regarding the timing of surgery, we offer surgical treatment in patients with failed medical therapy (with maximum oral opioids along with enzyme supplementation) and endoscopic therapy, development of locoregional complications, or suspicious of malignancy.

**Fig. 1:**
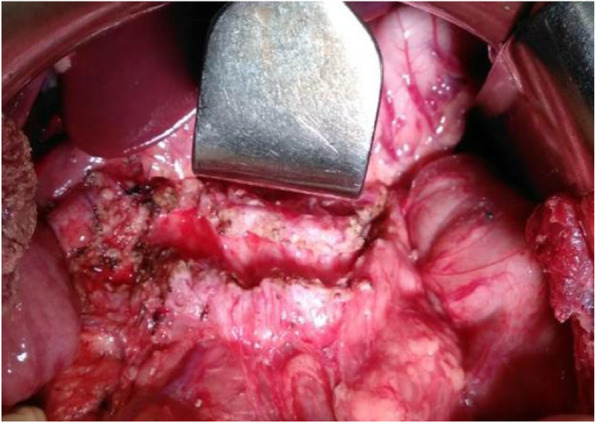
Frey’s picture- Opening of Main pancreatic duct (tail to head) with coring in head region

**Fig. 2:**
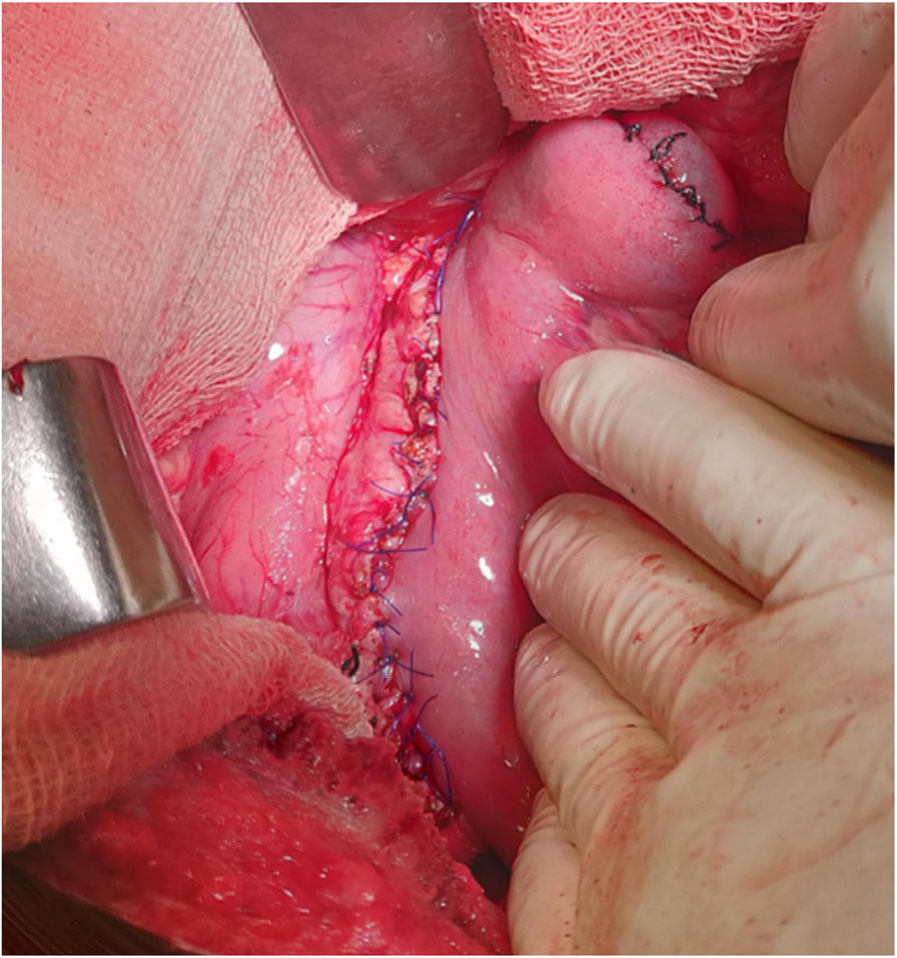
Frey’s intraoperative picture - completed pancreatojejunostomy

Frey’s procedure was first described by Frey and Smith in 1987 which is a hybrid procedure that includes resection of the head of the pancreas anterior aspect (coring) combined with drainage of the MPD using longitudinal pancreatojejunostomy [9]. Surgical procedure entails midline laparotomy with a careful assessment followed by ligation of right gastroepiploic vessels and full exposure of the anterior surface of the pancreas from head to tail (Fig. 1). After confirming the dilated MPD by fine-needle puncture or occasionally by ultrasonography (in difficulty), MPD is fully incised from anterior aspect approaching from tail up to head. Anterior branch of the gastroduodenal artery is ligated and hemostatic suture placed over, maximum coring (excision) of pancreatic parenchyma in the head & uncinate region is done leaving a thin rim of tissue around duodenum. Stones must be cleared from duct and parenchyma in the head region along with all parenchymal calcifications. Excised tissue should be sent for histopathological examination to rule out malignancy. After this jejunum is dissected 20 cm distal to Treiz ligament and opened up longitudinally along the antimesenteric side, and longitudinal side-side pancreatojejunostomy (Fig. 2) is performed by nonabsorbable monofilament suture, followed by end-side jejunojejunostomy. Careful hemostasis is very important in the Frey’s procedure following which a single abdominal drain is kept. It is advised that to prevent recurrence, complete decompression of the pancreatic ducts in the head of the pancreas and full-length drainage of the MPD from the head to the tail is the most important part of the surgery.

CP was diagnosed based on history and imaging. Abdominal contrast-enhanced computed tomography (CECT) was routinely performed in all cases for diagnosis and characterization of the disease process. Magnetic resonance cholangiopancreatography (MRCP) was performed to further delineate ductal anatomy if CECT was inadequate. As the availability of endoscopic ultrasound (EUS) is new to our institute as well as country, EUS was not as part of routine investigation. Histopathological diagnosis of CP was confirmed postoperatively except in one case who underwent EUS and biopsy before preceding to surgery. Endoscopic resonance cholangiopancreatography (ERCP) and stenting were performed in that same case. Postoperative analgesia was given by epidural or parenteral route or both followed by oral analgesia usually from the third postoperative day. Third-generation cephalosporin was routinely used as prophylactic at time of induction, which later on continued for 3 days in the postoperative period. Further continuation of antibiotics depends on patient clinical status or features of sepsis or preexisting cholangitis. Deep vein thrombosis prophylaxis used in selected high-risk cases. All patients were encouraged for intensive spirometry in the postoperative period. Drain usually taken out after day three after the drain fluid amylase value along with clinical judgment and drain amount. Patients were discharged after assumption of oral diet, after removal of drain and being fully mobilized.

Clinical characteristics and short – and long – term results were evaluated. Preoperative variables include demographics, etiology, duration of symptoms, the intensity of pain, analgesic requirements, presence of diabetes mellitus or steatorrhea, presence of pseudocyst, biliary obstruction (jaundice), duodenal obstruction, pseudoaneurysm, prior treatment. Similarly, intraoperative findings like the presence of bulky head, ductal stones, calcifications, and any complications were confirmed as of prior imaging. Also, operative findings like MPD diameter, intraoperative blood loss and operative time noted.

The pain had been evaluated by Visual analog scale (VAS), analgesia group and Izbicki score. VAS had been defined as a scale of 0 to 100, where 0 indicated no pain and 100 indicated severe, unbearable, continuous pain. Analgesia group had been defined as class 1, acetaminophen or Nonsteroidal anti-inflammatory drugs (NSAIDS); class 2, acetaminophen and weak opioid combinations (like tramadol); and class 3, strong opiates (morphine, fentanyl, oxycodone, etc) as per the World Health Organization analgesic ladder [10]. The Izbicki pain score is a validated pain score specifically designed for chronic pancreatitis [11, 12]. It consists of four questions regarding frequency of pain, the intensity of the pain (VAS score), use of analgesics, and disease-related inability to work. Based on these question a pain score can be calculated ranging from 0 (no pain) to 100 (severe, debilitating pain). The exocrine pancreatic function was evaluated with the presence of steatorrhea which had been defined as more than three stools per day with a nauseating smell and greasy and pale appearance [13]. Similarly endocrine pancreatic function had been evaluated as presence of diabetes mellitus (DM) which had been defined based on fasting sugar (more than or equal to 126 mg/dl), postprandial sugar (more than or equal to 200 mg/dl), and or the level of glycosylated hemoglobin (HbA1C more than 7.5) and the need to treat diabetes mellitus with diet, oral hypoglycemic agents, or insulin.

Perioperative events had been defined as per Clavien Dindo classification system of surgical complications [14]. Major complications were defined as events occurring within 30 days requiring intervention (IIIa – under local anesthesia, IIIb – under general anesthesia), intensive care organ support (IVa – single organ, IVb – multiorgan) and death (V). Also, the number of postoperative hospital stay was noted. The peripancreatic drainage fluid was collected and measured and the amylase level was monitored postoperatively to define the occurrence of any postoperative pancreatic fistula (POPF) in accordance with the definition of the international study group of pancreatic surgery (ISGPS) [15].

All patient had been followed up for a minimum of 3 months. Long term follow-up for a minimum of 12 months was available in 21 patients. None of the patients was lost to follow up. Though follow up interval is not uniform as many patients belong to rural distant areas and they intended to follow up depending on suitability, information regarding postoperative status was possible on phone calls. Pain intensity along with analgesic requirement was evaluated again with VAS score and Izibcki score on each follow-up along with the development of new-onset DM or steatorrhea. Any readmission or intervention needed in the postoperative period were also noted and occurrence of any postoperative events was managed as per institutional practice.

### Statistical analysis

Statistical analyses were performed using *Statistical Package for the Social Sciences* (SPSS) version 24.0 software (SPSS Company, Chicago, IL, USA). Quantitative data were expressed as mean ± standard deviation or median (whenever applicable) and range. Statistical difference between preoperative and postoperative pain scores was examined using the paired t-test. The Chi-square test or Fisher’s exact test were used to compare categorical variables, as appropriate. *P* values of < 0.05 were considered statistically significant.

## Results

Over the defined period, a total of 26 cases of CP underwent Frey’s procedure out of a total of 38 CP cases at TUTH.

### Patients

Table 1 shows the clinicopathological characteristics of the 26 patients categorized in tropical versus non-tropical CP (male, *n* = 14, 53.9%; female, *n* = 12, 46.2%; mean age, 27.9 ± 8.1 years, range 17–52 years). Females were predominant in tropical CP group as compared to male in non-tropical CP. Except the gender difference, there was no significant difference in between the groups (tropical versus non-tropical) in regards to age, preoperative BMI, preoperative VAS score, preoperative Izbicki pain score, preoperative diabetes and preoperative indigestion. Though, the median duration of symptoms was shorter in tropical group as compared to non-tropical group, it was not statistically significant difference. Three patients (11.5%) were smoker while six patients (23.1%) were regular alcohol consumer. Etiology of CP were alcoholic (*n* = 6, 23.1%) and nonalcoholic (*n* = 20, 76.9%). Out of nonalcoholic, the majority were classified as tropical CP (n = 13, 50%), recurrent biliary in one case while remaining were idiopathic (*n* = 6, 23.1%). All patients had preoperative CECT while MRCP was performed only in five cases. EUS and ERCP were performed in only one patient who had undergone endoscopic stenting for pain relief.

**Table 1:** Clinicopathological Characteristics (*n*=26)

	All (*n*=26)	Tropical CP (*n*=13)	Non-tropical CP (*n*=13)	*p*-value
Age (years)	26.5 (17-52)	26 (20-38)	27 (17-52)	0.925
Sex (M/F)	14/12	4/9	10/3	**0.018**
Duration of symptoms (months)	12 (4 -120)	12 (4-120)	24 (4-72)	0.943
Preoperative BMI (kg/m2)	21.3 ± 2.2	21.6 ± 2.6	21.0 ±1.9	0.547
Preoperative VAS score	70 ± 15.5 (30-90)	73.6 ± 11.2	66.2± 18.5	0.212
Preoperative Izbicki pain score	53.4 ± 17.6 (17.5-85)	56.5 ± 17.6	50.3 ±17.9	0.377
Preoperative Indigestion	2 (7.7%)	2 (15.4%)	0	0.480
Preoperative DM	6 (23.1%)	3 (23.1%)	3 (23.1%)	1.000

Categorical data expressed in number (%), & continuous data in mean ± SD or median (range)

Bold data is significant to *p*-value <0.05

*BMI* Body mass index, *VAS* Visual analogue score, *DM* Diabetes mellitus

Similarly, Table 2 describes the indication of surgery along with preoperative therapy. As part of an indication of surgery, intractable pain refractory to medical management were present in all cases. However, three cases (11.5%) had developed pseudocyst additionally and one case (3.8%) presented with pseudoaneurysm of the gastroduodenal artery who underwent coil embolization before undergoing surgery. Also, pseudocyst drainage was performed in two cases (7.7%) before surgery by USG guided percutaneous drainage. Regarding chronic pain management, type of analgesia class was class I (n = 1, 3.8%), class II (*n* = 8, 30.8%) and class III (*n* = 17, 65.4%). The type of analgesia was based on the maximum level of analgesia used at any point of life after being diagnosed as CP. Average preoperative VAS pain score was 70 ± 15.5 (30–90) while the average preoperative Izbicki score 53.4 ± 17.6 (17.5–85). Diabetes mellitus was present in six cases (23.1%) while features of indigestion were evident in two cases (7.7%). No patients had jaundice, features of biliary obstruction or duodenal obstruction in our series.

**Table 2:** Indications of surgery & Preoperative Therapy (*n*=26)

Indication of surgery	
Intractable pain	22 (84.6%)
Intractable pain with Pseudocyst	3 (11.5%)
Intractable pain with Pseudoaneurysm	1 (3.8%)
Treatment before Frey procedure	
Pancreatic stenting (ERCP)	1 (3.8%)
Pseudocyst drainage	2 (7.7%)
Coil embolization of pseudoaneurysm	1 (3.8%)

Data expressed in number of patients (%)

*ERCP* Endoscopic retrograde cholangiopancreatography

### Perioperative details

All relevant intraoperative details are shown in Table 3. All patients had MPD diameter more than 7 mm with an average of 10.0 ± 2.5 (range, 7–16). All patients had a bulky head. Multiple ductal stones were found in 24 cases (92.3%) and parenchymal calcification were evident in 25 cases (96.2%). Three patients received a blood transfusion in the postoperative period. No additional procedure was performed.

**Table 3:** Intraoperative details (*n*=26)

MPD diameter (mm)	10.0 ± 2.5 (7-16)
Ductal stones (multiple)	24 (92.3%)
Parenchymal calcification	25 (96.2%)
Bulky head	26 (100%)
Operation time (minutes)	242.7 ± 36.9 (200-360)
Operative blood loss (ml)	196 ± 78.4 (100-400)

Categorical data expressed in number (%), & continuous data in mean ± SD or median (range)

*MPD* Main pancreatic duct

Similarly, perioperative outcomes are shown in Table 4. Postoperative major complications over 30 days (Clavein Dindo grade IIIa and above) were seen in three cases (11.5%). One patient was readmitted with deep surgical site infection who underwent secondary suturing under local anesthesia on 26th postoperative days. Similarly, one patient developed a burst abdomen on the 9th postoperative day in the hospital who underwent laparotomy and retention suturing under general anesthesia. There was one (3.84%) mortality over 30 days duration. The mortality was of an alcoholic CP discharged on the 6th postoperative day from the hospital but presented at a local hospital with severe hematemesis and attempted to manage in the intensive care unit of the local hospital with multiple blood transfusion succumbed to death before presenting to our institute. Possible cause of death might be upper GI bleed due to variceal rupture as the same patient had a cirrhotic appearance of the liver. Considering the perioperative outcomes, tropical CP had two major complications while non-tropical CP had only one major complication in the form of mortality. Maximum drain amylase on or after day 3 was average of 59 U/L (range, 9–390). No patients developed clinically relevant POPF while one patient had a biochemical leak. Regarding the specific type of complications, six patients had surgical site infection (two required intervention as described above and four was managed with dressing and antibiotic upgrade and three had postoperative pneumonia (managed with an upgrade of antibiotics and supportive care). Histopathology of the cored specimen confirmed the diagnosis of CP and no evidence of malignancy were found.

**Table 4:** Perioperative outcomes (N/%) (*n*=26)

Perioperative blood transfusion	3 (11.5%)
Maximum Drain amylase (day 3 or above)	59 (9-390)
Major complications over 30 days (Clavein-Dindo Grade IIIa and above)	3 (11.4%)
Mortality over 30 days	1 (3.8%)
Postoperative hospital stay (days)	7 (4-18)
Readmission in 30 days	1 (3.8%)
Relaparotomy	1 (3.8%)

Data expressed in number of patients (%) & continuous data in mean ± SD or median (range)

### Short – and long – term outcomes

Table 5 shows the short – and long – term outcomes of patients. Median duration of follow up was 17 months (range, 3–38 months). A minimum follow up of 3 months was possible in all survival (25 cases) without lost to follow-up. Similarly, follow up of more than 12 months were analyzed in 21 cases. Two patients were readmitted for pain abdomen and managed conservatively with intravenous analgesics and discharged. One patient developed new-onset diabetes mellitus while no new cases of steatorrhea were evident.

**Table 5:** Short and long term outcomes over follow-up period

Follow up duration (months)	17 (3-38)
Readmission (25)	2 (8%)
Postoperative VAS score (25)	24.4 ± 14.2 (10-60)
Postoperative Izbicki score at 3 month follow up (25)	10.7 ± 11.4 (2.8-47.5)
Postoperative Izbicki score at 12 month follow up (21)	9.9 ± 9.0 (2.8- 36.25)
Postoperative diabetes mellitus (25)	7 (26.9%)
Postoperative indigestion/ steatorrhea (25)	2 (7.7%)

Categorical data expressed in number (%), & continuous data in mean ± SD or median (range)

*VAS* Visual analogue score

Comparison of pain score and the function of the pancreas is described in Table 6. Pain intensity scores (VAS score, Izbicki score at 3 months and Izbicki score at 12 months) were significantly decreased in postoperative as compared to preoperative (*p*-value < 0.01). Also, there was no significant worsening of endocrine (DM) or exocrine (steatorrhea) function.

**Table 6:** Comparison of pain and pancreatic function before and after surgery

	Preoperative	Postoperative	*p* value
Pancreatic function (25) (N/ %)			
DM	6/25 (24%)	7/25 (26.9%)	0.327
Steatorrhea	2/25 (8%)	2/25 (8%)	1
Pain score (Mean ± S.D)			
VAS score (25)	70.0 ± 15.8	24.4 ± 14.17	**<0.01**
Izbicki pain score at 3 months (25)	53.4 ± 18.0	10.7 ± 11.36	**<0.01**
Izbicki pain score at 12 months (21)	54.7 ± 19.33	9.9 ± 9.03	**<0.01**

Categorical data expressed in number (%), & continuous data in mean ± SD or median (range)

Bold data is significant to *p*-value <0.05

*DM* Diabetes mellitus, *VAS* Visual analogue score

## Discussion

Owing to the geographical difficulties, and financial constrain, providing one time treatment which is long lasting along with acceptable perioperative morbidities, surgery is definitely the cornerstone of management of CP in counties like Nepal. It is well known that Frey procedure can be very helpful in patients with CP where the benefit of one time treatment outweighs the perioperative complications along with the uncertainty regarding multiple need of endoscopic therapy along with financial burden. The results of our study suggests that Frey’s procedure is safe and causes relief of symptoms in patients with CP. Ninety-two percent of patients were pain-free with significantly lower pain scores postoperatively while major complications developed in 11.5% cases without worsening of exocrine and endocrine functions. So Frey’s procedure can be considered a choice of treatment of CP in countries like Nepal.

Alcohol is the most common etiology of CP worldwide. However, there is a form of idiopathic early-onset CP prevalent in tropical countries like India and the southern part of Nepal popularly known as tropical pancreatitis. Tropical Pancreatitis (TP) is a juvenile form of chronic calcific, nonalcoholic pancreatitis [16]. It is seen almost exclusively in developing countries of the tropical world. Contrary to other forms, TP is characterized by younger-onset, presence of large intraductal calculi, accelerated course of the disease, and high susceptibility to pancreatic cancer. The exact pathogenesis is largely unknown, however, various genetic, nutritional and inflammatory factors may have a role [17]. Tropical pancreatitis had female predominance while non tropical had male predominance.

Regarding pain score, both VAS and Izbicki had higher mean values in tropical pancreatitis in comparison to non-tropical and hence tropical pancreatitis got operated earlier than non-tropical. Mechanism of pain is poorly understood but traditionally is caused by pancreatic ductal hypertension and pancreatic parenchyma hypertension. Newer theories include activation of intrapancreatic nociceptors, hypertrophy, and inflammation of intrapancreatic nerves and abnormal pain processing in the central nervous system [18]. Long-standing complications can be pseudocysts; common bile duct stricture causing obstructive jaundice; duodenal stenosis causing gastric outlet obstruction; portal vein thrombosis; splenic vein thrombosis with fundal varices; pseudoaneurysm affecting the splenic, hepatic, gastroduodenal, and pancreaticoduodenal arteries; and pancreatic ascites. Also, there is a higher risk of pancreatic adenocarcinoma and is more often in patients with early-onset CP like in hereditary and tropical [19].

The current standard of management of painful CP is multidisciplinary step-up approach as published in HaPanEU guideline in 2017 [8]. Step up approach includes the first step which is conservative treatment including alcohol abstinence, cessation of smoking, analgesics, enzymatic supplementation along with dietary advice. Despite this, if pain persists, endoscopic treatment should be advised in the form of drainage and or stenting of pancreatic duct and removal of ductal stones. The final step is the surgical treatment if medical and endoscopic therapy fails. Nutrition and lifestyle modification along with alcohol and smoking cessation are key components of a successful management plan. The problem with long-term use of opioids is tolerance and dependence and should be best avoided [3]. Interventional endoscopy is an integral component of a multidisciplinary approach with good success rates especially in patients with disease confined to head of the pancreas and long term success depends on the degree of ductal clearance.

Major complications were seen in 11.5% and one patient (3.8%) had mortality in our series and is compared with various published reports of morbidity (7.5–22.0%) and mortality (0–3.2%) [20, 21]. None patient developed clinically relevant POPF and no cases had post pancreatic hemorrhage (PPH). These results confirmed that the Frey’s procedure is safe. The improved perioperative outcome is likely due to the development of better perioperative care, proper patient selection and technically sound surgical expertise with dedicated four gastrointestinal surgical units treating all kinds of benign and malignant gastrointestinal diseases.

The intensity of pain as assessed by VAS score and Izibcki score had decreased significantly following Frey’s. In our study only two patients require readmission due to exacerbation of pain and were managed with intravenous analgesics and rest of patients were comfortable without the need of opioids analgesia in the postoperative period over a median follow up of 17 months. Results of pain relief are 92% compared to other reports [21, 22]. Frey’s procedure is effective for preventing acute painful exacerbations. Our series had developed the new onset of diabetes mellitus in one case (3.8%) which is better than previous reports of 10–20% of patients after the Frey’s procedure for CP [21]. So Frey’s procedure is safe in terms of preserving endocrine function postoperatively. Pancreatic enzyme supplementation was given in most of the patients following surgery and none developed any evidence of steatorrhea [23]. However previous reports showed exocrine insufficiency in 60–90% patients following surgery [21]. Pancreatic enzyme replacement therapy prevents the development of symptomatic pancreatic exocrine insufficiency after the Frey’s procedure.

ESCAPE (Early surgery versus optimal current step-up practice for chronic pancreatitis) trial conducted by the Dutch Pancreatitis Study Group will give a clear answer to the optimal timing of surgery in CP [12]. This randomized trial entails two treatment arm either surgery within 6 weeks after randomization or the current step-up approach including endoscopy. Though not published yet, preliminary results presented at United European Gastroenterology Week 2018, shows that early surgery outperforms conservative treatment and endoscopy [2].

Frey involves local resection of the head of the pancreas so it prevents the inadequate decompression of the pancreatic ducts in the head of the pancreas unlike the Partington–Rochelle pancreatojejunostomy. About 90% long term pain relief has been reported after the Frey’s procedure [21, 22]. Also Frey’s is less morbid as compared to more extensive resections like Beger and pancreatoduodenectomy. But one disadvantage of Frey’s is that a rim of pancreatic tissue of the pancreatic head with active disease is left in place so that might cause recurrence of pain. However, in patients with CP without diffuse dilation of the MPD and a possibility of malignancy, Frey’s procedure should be avoided. For such patients, pancreatic head resection such as PD should be performed.

Rajendra VKP reported the effectiveness of Frey’s procedure in tropical pancreatitis from India in reducing pain along with significant improvement in health-related quality of life [24]. Long term follow-up of Frey’ procedure from a center of Japan had reported effective pain relief and also is a safe technique for the management of CP [25].

So Frey’s procedure could be the standard operation for CP patients with dense calcification or stones in the head of that pancreas and dilation of the MPD and can be performed for almost all patients with CP and dilation of the MPD without suspicious of malignancy. The Frey’s procedure is safe and pancreatic endocrine function is preserved. However, a proper selection of patients is the most integral part of achieving good results.

### Limitations

This study has some limitations. First being retrospective in nature and this review was only of the patients operated. So other patients under medical management and endoscopic treatment were not evaluated. Also, outcomes have not been compared between Frey’s and other procedures. Though follow-up was available in all cases, fix interval of follow-up were not possible. Also, long term follow-up involving a larger sample size is needed to conclude the best surgical procedure. Median follow up of 5 years will help to assess the long term outcome of Frey’s procedure in making it the choice of treatment.

## Conclusion

Our early experiences suggests that Frey’s procedure can be a safe option for patients with CP, with acceptable perioperative morbidity with adequate pain relief without worsening of pancreatic endocrine and exocrine function. However, further prospective controlled studies with long-term follow-up are needed to make definitive conclusion.

## Data Availability

The datasets used and/or analyzed during the current study are available from the corresponding author on reasonable request.

## Abbreviations

BMI: Body mass index
CECT: Contrast enhanced computed tomography
CP: Chronic pancreatitis
DM: Diabetes mellitus
ERCP: Endoscopic resonance cholangiopancreatography
ESCAPE: Early surgery versus optimal current step up practice for CP
EUS: Endoscopic ultrasound
ISGPS: International study group of pancreatic surgery
MPD: Main pancreatic duct
MRCP: Magnetic resonance cholangiopancreatography
PD: Pancreatoduodenectomy
POPF: Postoperative pancreatic fistula
SPSS: Statistical Package for the Social Science*s*
TP: Tropical Pancreatitis
TUTH: Tribhuvan University Teaching Hospital
VAS: Visual analogue scale
